# Patterns of preschool children’s screen time, parent–child interactions, and cognitive development in early childhood: a pilot study

**DOI:** 10.1186/s40814-023-01266-6

**Published:** 2023-03-14

**Authors:** Jasmine Rai, Madison Predy, Sandra A. Wiebe, Christina Rinaldi, Yao Zheng, Valerie Carson

**Affiliations:** 1 Faculty of Kinesiology, Sport and Recreation, University of Alberta, 8840-114 St., Edmonton, AB T6G 2H9 Canada; 2 Faculty of Arts – Department of Psychology, University of Alberta, Edmonton, AB Canada; 3 Faculty of Education – Department of Educational Psychology, University of Alberta, Edmonton, AB Canada

**Keywords:** Screen time, Preschool children, Parent–child interactions, Cognitive development

## Abstract

**Background:**

The primary objective of this study was to explore the feasibility of a virtual study protocol for a future longitudinal study, including recruitment, study measures, and procedures. The secondary objective was to examine preliminary hypotheses of associations, including 1) the correlations between total duration and patterns of screen time and cognitive development, and 2) the differences in quality of parent**–**child interactions for two screen-based tasks and a storybook reading task.

**Methods:**

Participants included 44 children aged 3 years and their parents from Edmonton, Alberta and surrounding areas. Children’s screen time patterns (i.e., type, device, content, context) were parental-reported using a 2-week online daily diary design. Children’s cognitive development (i.e., working memory, inhibitory control, self-control, and language) was measured virtually through a recorded Zoom session. Parent**–**child interactions during three separate tasks (i.e., video, electronic game, and storybook reading) were also measured virtually through a separate recorded Zoom session (*n* = 42). The quality of the interactions was determined by the Parent–Child Interaction System (PARCHISY). Descriptive statistics, Intra-class correlations (ICC), Spearman’s Rho correlations, and a one-way repeated measures ANOVA with a post-hoc Bonferroni test were conducted.

**Results:**

All virtual protocol procedures ran smoothly. Most (70%) participants were recruited from four 1-week directly targeted Facebook ads. High completion rates and high inter-rater reliability in a random sample (Diary: 95% for 13/14 days; Cognitive development: 98% 3/4 tests, ICC > 0.93; Parent–child interactions: 100% for 3 tasks, Weighted Kappa ≥ 0.84) were observed for measures. Across cognitive development outcomes, medium effect sizes were observed for five correlations, with positive correlations observed with certain content (i.e., educational screen time) and negative associations observed for total screen time and certain types (show/movie/video viewing) and contexts (i.e., co-use). Medium and large effect sizes were observed for the differences in parent–child interaction quality between the three tasks.

**Conclusions:**

The virtual study protocol appeared feasible. Preliminary findings suggest it may be important to go beyond total duration and consider type, content, and context when examining the association between screen time and cognitive development. A future longitudinal study using this virtual protocol will be conducted with a larger and more generalizable sample.

**Supplementary Information:**

The online version contains supplementary material available at 10.1186/s40814-023-01266-6.

## Key messages regarding feasibility

***•**** What uncertainties existed regarding the feasibility?* The COVID-19 pandemic has introduced new challenges to collecting data in young children and their parents. The feasibility of recruiting for and conducting a virtual study protocol to inform a future longitudinal study that examines preschool children’s screen time, parent–child interactions and cognitive development is unknown.

***•**** What are the key feasibility findings?* No major issues arose for any of the virtual protocol procedures. A total of 47 parent–child dyads were recruited to participate in this pilot study and 44 parent–child dyads completed study measures. Four weeks of targeted Facebook ads yielded the most (70%) participants. On average, 95% of participants provided 13 (*n* = 6) or 14 (*n* = 36) days of complete diary data. Additionally, 98% of participants completed three (*n* = 3) or four (*n* = 38) of the cognitive tests. Finally, 100% of participants completed the parent–child interaction tasks. In a randomly selected sample (20%), high inter-rater reliability across cognitive development tests (ICC > 0.93) and parent–child interaction tasks (Weighted Kappa ≥ 0.84) were observed.

***•**** What are the implications of the feasibility findings for the design of the main study?* The virtual study protocol, including recruitment and measurement, was feasible and reliable in 3-year olds and their parents. Retention of parent–child dyads for the longitudinal study remains unknown. Based on these findings a future larger longitudinal study will be conducted using this virtual protocol.

## Background

Preschool children (3–4 years) experience rapid growth and development of cognitive development [5, 20, 46]. In particular, in the language and executive functioning domains children rapidly develop symbolic thought [16, 35] and other skills such as working memory and self-control during this time period [11, 17, 34]. However, children’s cognitive development is dependent on their environment and experiences [43]. Evidence suggests screen time, especially television viewing, is either unfavorably associated with or provides no benefits for cognitive development [36]. Therefore, it is troubling that many preschoolers exceed national and international screen time recommendations (i.e., < 1 h/day) [10, 28]. Increased screen time in this age group is thought to be replacing activities that are beneficial for cognitive development [3].

Given that so many preschoolers exceed screen time recommendations [10, 28], it is important to understand the patterns of screen time that contribute to increased exposure in this age group. For the present study, the patterns of screen time include total duration as well as the type of screen time (e.g., watching television, playing a game), the device being used (e.g., tablet, smartphone), the content (e.g., education preschool, educational school age, entertainment, adult, other), and the context (e.g., co-viewing). While there is growing recognition in the importance of understanding the quality and context of screen time [21, 26, 48], the majority of studies in the current literature have only focused on total duration or frequency. This highlights an important gap because a recent meta-analysis reported that the total duration of screen time was negatively associated with language development in children, but educational content and co-viewing were positively associated with language development [30, 31]. However, the majority of studies in this review focused on television viewing, and little is known about the effects of mobile screen devices (e.g., smart phones, tablets) on cognitive development. Therefore, considering the patterns of screen time, including mobile devices, can provide important insight into the impacts of screen time on children’s cognitive development.

In terms of screen time context, co-use of mobile screen devices may provide opportunities for parent**–**child interactions and there is strong evidence to indicate that high-quality interactions are important for children’s cognitive development [25, 29]. There is some evidence that the quality of parent**–**child interactions differs depending on the type of task [4, 27, 39, 41]. For example, one study found that in comparison to engaging in toy play or watching television, mothers were more sensitive and structuring during joint gaming on a tablet [41]. Additionally, more hostility was observed by mothers during the toy play, in comparison to the other tasks [41]. Further research is needed to better understand if the quality of parent**–**child interactions differs depending on the type of screen device being used and how much screen time involves co-use with parents versus independent screen time.

To address current evidence gaps regarding screen time and cognitive development in early childhood, we conducted a pilot study in a sample of 3-year-olds and their parents. The primary objective of this study was to explore the feasibility of a virtual study protocol including recruitment, study measures and procedures due to the COVID-19 pandemic. Specifically, for recruitment, the willingness of families to participate in a virtual study on screen time and cognitive development and the best sources of recruitment were examined, for study measures, compliance rates and inter-rater reliability were examined, and for procedures, the ability of the virtual protocol components to all work together and run smoothly was examined [18]. The secondary objective of this study was to examine preliminary hypotheses of associations, including 1) the correlations between total duration and patterns of screen time and cognitive development, and 2) the differences in quality of parent**–**child interactions for two screen-based tasks and a storybook reading task.

## Methods

### Participants and procedures

Participants included 47 parents and their children aged 3 years from Edmonton, Alberta and surrounding areas. A combination of registries and online advertising was used to recruit participants between August and December 2020. A screening interview was conducted with each interested family to determine eligibility. Inclusion criteria included children being 36**–**48 months old and living with their main caregiver in or around Edmonton, Alberta. Families were excluded if children were born preterm (gestational age of < 37 weeks) or underweight (< 2500 g) or if children had been diagnosed with a neurological or psychiatric disorder that affects neurocognitive development. The screening form included 15 different illnesses or medical conditions (e.g., autism spectrum disorder, attention deficit hyperactivity disorder). Families were also excluded if parents could not speak or read English fluently or if they did not have a laptop, computer, or tablet with a camera and microphone. During the screening interview, families were also asked which person(s) spends the most time with their child when they are engaging in activities like screen time and reading. The person identified was instructed to complete study items. If two people equally spend time with the child during these activities, both caregivers were invited to participate in separate parent–child interaction sessions, outlined in the next paragraph. Additionally, during the screening interview or one of the online sessions, families were asked how they found out about the study.

Data was collected virtually through separate Zoom sessions and REDcap, an electronic data capture tool [22], to ensure safety during the COVID–19 pandemic. Further details on the measures are provided in the next section. A consent form and questionnaire were sent once participants were determined as eligible to complete online via REDcap before the first Zoom session. During the first session, parent**–**child interactions were assessed using three tasks: (a) watching one of two television shows via YouTube, (b) playing one of two electronic games, and (c) reading one of two eBooks. Each of the tasks took approximately 5 to 8 min to complete. Each session took approximately 25**–**30 min to complete. The order of the tasks was randomized, and participants were randomly assigned to 1 of the 2 options for each task. In the case that the child was familiar with the assigned option, the alternate option was used (*n* = 3). If two people identified themselves as primary caregivers (*n* = 1; mother and father), the second caregiver was sent a consent form via REDcap and a separate Zoom session (7–10 days after the second session) was scheduled for parent–child interactions. The second option for each task was used in this session. The day after the first session, parents began the completion of a 2-week online daily diary of screen time use via REDcap. The second virtual session took place approximately one week (i.e., 7–10 days) after the first session. During this session, children’s cognitive development was assessed using four different tests in the following order: expressive vocabulary test, head toes knees shoulders (HTKS) test, the word span test, and a snack delay test. These tests were selected because they capture key domains of cognitive development (memory, executive functioning, and language), the tests have been validated in preschool children [6, 24, 37, 49], and they are feasible to administer virtually. If children were unable to complete all four tests during the second Zoom session (*n* = 15), a third Zoom session was scheduled approximately 7–10 days after the second session. Parents received an electronic gift card up to $48 after they were done participating in the study. Specifically, parents received $20 for the 2–3 virtual meetings and a maximum of $28 ($2 per daily entry) for the 2 week daily diaries. The University of Alberta Research Ethics Board provided ethics approval for the study and all participating parents provided written informed consent via REDcap. Additionally, all parents provided verbal consent for the recording of Zoom sessions.

### Measures

#### Screen time patterns

To measure screen time patterns, parents completed a 2-week online daily diary survey. They recorded all morning, afternoon, and evening sessions of children’s screen time use each day. A session was defined as any time children engaged in screen time. Morning sessions were defined as any time children engaged in screen time starting before 12:00 PM, afternoon sessions were defined as any time children started engaging in screen time between 12:00**–**4:59 PM, and evening sessions were defined as any time children started engaging in screen time that started at 5:00 PM or later. Additional sessions were recorded if children engaged in screen time at multiple times during the day (e.g., morning session 1, morning session 2, afternoon session 1, etc.). For each session, parents recorded what time the session began and ended (e.g., 1:00**–**1:20 pm) and what type of device was used (i.e., television, tablet, smartphone/cell phone, computer, laptop, video game console, or other). Parents also recorded the type of screen time (i.e., show/movie/video, electronic game, communication, or other), the content (i.e., program/game name) and the context (e.g., if and who watched/played with the child). Parents were given the option to specify if they responded “other” when recording the device and type of screen time. For time, content, and context, response options were open ended. Three members of the research team developed content categories and definitions based on various categories and definitions provided by previous research [2, 32]. The specific categories included entertainment, education preschool, education school aged, adult, and other. Definitions for these categories are provided in Table 1. One research team member categorized the program/game name provided by parents into one of these categories by reviewing program episodes or games and their descriptions. The context was categorized into co-use with an adult (e.g., parent, other relative, caregiver), co-use without an adult (e.g., sibling, other child) and no co-use. Only co-use with an adult was included in this study because we wanted to examine parent**–**child interactions. During the first Zoom session, parents were asked if children spent time in care other than that of the parent. If parents responded yes (*n* = 16), they were emailed a copy of the other caregiver dairy that they could give to the other caregivers in order to capture any screen time that took place under their supervision. The diary also included questions about whether it was a typical day, as defined by the parent, and any factors that could have impacted the day (e.g., childcare, sleep, illness). If parents identified a day as atypical because it was a weekend day, it was coded as a typical day. Daily diaries have been shown to be more accurate than global time estimates because they do not rely on the participant to recall events from the entire day but rather focus on discrete time periods [47].

**Table 1:** Children’s screen time content definitions

Definition
Education preschool-aged • Coherent and integrative narrative (show/movie/video only) • Language/topic appropriate for preschool-aged child • Labelling or finding objects and/or speaks directly to the child throughout the program • May provide opportunities to respond verbally • May be labelled as a show/game for preschool-aged children
Education school-aged • Coherent and integrative narrative (show/movie/video only) • Language/topic appropriate for school-aged children • May be labelled as a show/game for school-aged children
Entertainment • Non-adult content that does not involve labelling or finding objects and/or speaks directly to child • May not have a coherent and integrative narrative • Does not provide opportunities to respond verbally
Adult • Adult appropriate language and topics, including sports • May be labelled as a show/game for adults
Other • Non-adult content that does not involve labelling or finding objects and/or speaks directly to child throughout programming • Not enough detail provided by parent (e.g., only station/network provided) • Activity based programming (e.g., yoga)

#### Cognitive development

Working memory was assessed using the forward and backward span phases of a word span test [6, 45]. The researcher read a sequence of words and the children were asked to repeat the words back in the same order (forward span phase) or in reverse order (backward span phase). Each block in the forward and backward span phases had three trials unless the child responded correctly to the first two trials, at which point the third trial was skipped and they moved onto the next block. If all three trials were incorrect in a block, the phase was terminated. The trials began with two-word sequences and increased in length for each subsequent block. The outcome variable for this test was a final score that was an average of all the trials attempted for the forward and backward span phases [45]. Possible scores ranged from 0**–**5 and a higher score indicates better working memory. The words used for the trials included age appropriate, monosyllabic nouns that were different enough to minimize semantic or phonological interference (e.g., cake, stick).

The HTKS test was used to measure children’s inhibitory control, where the child had to inhibit a dominant response [37]. The children were asked to play a game where they had to do the opposite of what the researcher said. For example, the researcher asked the child to touch their head in which case the child had to touch their toes and vice versa. In a second, advanced phase trials were added where the researcher asked the child to touch their knees and they had to touch their shoulders. The outcome variable was the sum of the first 6 practice trials and 20 test trials. For each trial, children received a score of 0,1 or 2 if they responded incorrectly, self-corrected, or correctly, respectively. Possible scores ranged from 0**–**52 and a higher score indicates better inhibitory control.

Language was assessed using the expressive vocabulary test in the iPad-based Early Years Toolkit [24]. The children were shown pictures on a slideshow that was screen shared via Zoom. Researchers scored the test using an iPad with the app that displayed the pictures matching the slideshow shown to the children. Children named as many objects correctly as possible, with the words increasing in complexity as the test progressed. The game ended if the child incorrectly named 6 items in a row. The outcome variable for this test was a final score calculated based on the number of correct responses. Possible scores ranged from 0**–**45 and a higher score indicates better vocabulary. The expressive vocabulary test from the Early Years Toolkit has previously shown excellent reliability (Cronbach’s α = 0.92) and good convergent validity (*r* (84) = 0.60, *p* < 0.001) when compared to existing measures [24].

Self-control was assessed using a modified snack delay test in which children had to maintain a fixed posture for four minutes to gain a reward [49]. The child was instructed to “sit still and silent like you’re frozen” with their hands placed on the table. The parent then placed six small snacks under a transparent cup or container within reach of the child on the table in front of them. When the researcher rang the bell, the child could eat the snack. Before the actual trial, a practice trial was conducted where the child had to wait 10 s before they could eat the snack. Throughout the 4-min trial, parents and the researcher pretended to be otherwise engaged and executed prompts at set intervals to distract the child. The snack delay task was broken into 5 s epochs that were scored out of 3 based on hand movement, body movement and speaking. Children lost a point if they moved their hands, moved their bodies or spoke during each 5 s epoch. The outcome variable for this test was a final score based on these movement and speaking categories. Possible scores ranged from 0**–**144 and a higher score indicates better self-control. The snack delay has previously shown good test–retest reliability of *r* > 0.80 [40].

#### Parent–child interactions

Parent**–**child interactions were examined during three tasks. Parents and children were instructed to read a storybook (*Pete the Cat’s First Day of Preschool* or *Bears and a Birthday*), watch a short video (*Paw Patrol Mighty Pups: Pups vs. The Super Sonic Sound System* or *Peppa Pig: The Market*) and play an electronic game (*Curious George Hide and Seek* or *peg* + *cat The Highlight Zone*) via either a tablet, laptop, or desktop computer. Tasks were standardized and functioned similarly across devices. Parent**–**child interactions during these tasks were recorded so that they could be coded afterwards.

The recordings of parent**–**child interactions were coded using the Parent–Child Interaction System (PARCHISY) [13, 14]. The PARCHISY consists of 18 items and uses a combination of codes for parent’s behaviour (i.e., positive affect, negative affect, verbalizations etc.), child’s behaviour (i.e., noncompliance, autonomy/independence etc.), as well as codes for dyadic interactions (i.e., reciprocity, conflict) [13, 14]. The PARCHISY scale was adapted from a 7-point Likert scale to a 5-point Likert scale for this study to allow for more consistency in coding for the specific tasks used in the present study. For example, for the positive effect (warmth) item, “one or two instances of positive affect” and “few/several instances of positive affect” were collapsed into one category. Additionally, “moderate amounts of positive affect – smiling, laughing for about half of interaction” and “positive affect for more than half the interaction” were collapsed into one category. As a result, the 5-point Likert scale included the following five categories: none, occasional displays, moderate amounts – approximately half of interaction, substantial amounts, and constant positive affect – all of affect is positive. Possible scores ranged from 0**–**90 and a higher total score represented better quality parent**–**child interactions [14, 15]. Any interactions related to technology (e.g., children needing help using a computer mouse or swiping on a tablet) was not included in the scoring. If two people completed the parent–child interaction tasks with the child (*n* = 1), an average parent–child interaction quality score was calculated. High inter-rater reliability for the PARCHISY (Cronbach’s α ≥ 0.80) has previously been reported [19].

#### Demographic information

Demographic information from participants was collected using a parent questionnaire. Parents were asked to report their child’s sex, race/ethnicity, the number of siblings (including stepsiblings) that live in the home with them, and the number of hours/week their child spent in childcare. Parents also reported their age, sex, gender identity, household income, and education level.

#### Statistical analyses

Statistical analyses were conducted using STATA 15 and SAS 9.4 software. Descriptive statistics were calculated for compliance rates, parents’ and children’s demographic characteristics and total duration and patterns of preschool children’s screen time. For the cognitive development tests and parent**–**child interaction tasks, 20% of the sample was randomly selected and inter-rater reliability was calculated for two raters. Spearman ρ coefficients were used to determine whether there was an association between each screen time and cognitive development variable because the cognitive development test scores were not normally distributed. Next, partial Spearman ρ correlations were computed, adjusting for child age and parental education. These two variables were selected because they were the only demographic variables significantly correlated with one or more cognitive developmental outcomes. Due to the small sample size, we focused on effect size for interpretation: small, medium, and large effect sizes were defined as *r*_*s*_ = 0.10, *r*_*s*_ = 0.30 and *r*_*s*_ = 0.50, respectively [12]. To address the second objective, means and standard deviations for the quality of parent**–**child interactions were calculated, and a repeated measures ANOVA was used to determine whether parent**–**child interaction quality differed based on the type of task. The assumptions of normality and equality of variances were checked for the ANOVA. A Bonferroni post-hoc test was conducted to determine which tasks were significantly different and Cohen’s *d* coefficients were calculated to determine the effect size. Small, medium and large effect sizes were defined as *d* = 0.20, *d* = 0.50 and *d* = 0.80, respectively [12]. Finally, sensitivity analyses were conducted to examine patterns of children’s screen time use and to examine the association between children’s screen time use and cognitive development, when only diary days that parents reported being typical were included. Statistical significance was set at *p* < 0.05 for all statistical tests.

## Results

**Fig. 1:**
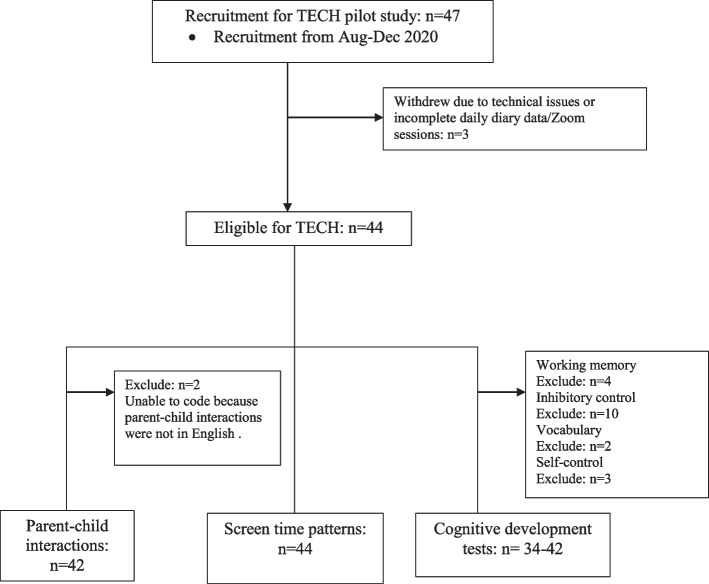
The TECH pilot study recruitment flow chart. Participants eligible for the TECH study completed a Zoom session examining parent–child interactions during 3 screen-based tasks. Parents were then asked to complete a 2-week daily screen time diary and report duration, type, device used, content and context. Approximately one week after completing the parent–child interaction task, children’s cognitive development was assessed, via a Zoom session, using 4 separate tests (i.e., working memory, inhibitory control, vocabulary and self-control)

A recruitment flow chart of the TECH study is provided in Fig. 1. Of the 47 parent**–**child dyads recruited for this study, two participants withdrew from the study due to technical problems and one did not complete daily diary surveys or Zoom meetings, leaving a sample of 44 parent**–**child dyads. No issues arose in meeting the target sample size of the pilot study (*n* = 40). In terms of the source of recruitment, 70% of eligible participants were recruited through four 1-week targeted (i.e., Edmonton parents of preschool children) paid Facebook ads, 12% from participant registries, and the remaining 18% were from word-of-mouth and other social media ads. For the targeted Facebook ads, a range of $30-$50 per week was used. The participant characteristics for parent**–**child dyads are presented in Table 2. Of the 44 parent**–**child dyads, two children were excluded from the analyses because the children primarily spoke a language other than English resulting in them not being able to complete the cognitive development tests and researchers being unable to code the parent–child interaction tasks. Additionally, children that did not attempt or complete a cognitive development test were excluded from that test (see Fig. 1). Of note for cognitive development outcome variables, few children (*n* = 3) progressed to the backward span phases of the working memory test. Additionally, all children were able to wait 4 min for the snack delay task, though there was variability in movement and quietness (see Table 2).

**Table 2:** Participant Characteristics

	**Mean/Category (SD/Percent)**
**Children’s Demographics**	***N* = 44**
Child Age (years)	3.5 (± 0.3)
Sex	
Male	20 (45.5%)
Female	24 (54.6%)
Siblings	
0	6 (13.6%)
1	23 (52.3%)
2 +	15 (34.1%)
Race/Ethnicity	
Caucasian	30 (68.2%)
Non-Caucasian	14 (31.8%)
**Children’s Cognitive Development**	
Working memory (Range: 0–5; *n* = 40)	0.9 (± 0.4)
Inhibitory control (Range: 0–52; *n* = 34)	16.6 (± 15.0)
Vocabulary (Range: 0–45; *n* = 42)	25.3 (± 6.0)
Self-control (Range: 0–144; *n* = 41)	75.8 (± 31.9)
**Parental Demographics**	
Relationship to Child	
Mother	43 (97.7%)
Father	1 (2.3%)
Parental Age (years)	35.2 (± 3.4)
Parental Education	
High school diploma or college/ trade certificate	8 (18.2%)
Bachelor’s degree	23 (52.3%)
Post-graduate	13 (29.6%)
Household income	
< $100 000	8 (18.2%)
$100 001—$150 000	25 (56.8%)
> $150 000	11 (25.0%)
Non-parental care (hours/week)	15.2 (± 17.5)

In terms of compliance rates for the measures, 95% of participants provided 13 (*n* = 6) or 14 (*n* = 36) days of complete screen time diary data. Parent feedback indicated that the diary was easy and quick to complete and that the online format was convenient. Additionally, 98% of participants provided data for three (*n* = 3) or four (*n* = 38) cognitive development tests. The inhibitory control test had the lowest completion rate (*n* = 34) out of the four tests, due to the number of trials in this test. Finally, all participants (100%) of parent–child dyads completed all three parent–child interaction tasks.

In terms of the inter-rater reliability of measures between two scorers, for working memory, high inter-rater reliability was observed for both the forward span and backward span phases (ICCs = 0.94, ICC = 1.00, respectively). High inter-rater reliability was also observed for the HTKS test and self-control tests, (ICC = 0.996, ICC = 0.96 respectively). Similarly, for the parent–child interaction tasks, a high inter-rater reliability was observed (Weighted Kappa ≥ 0.84).

The patterns of children’s screen time are presented in Table 3. Children spent on average 103.5 min/day (SD = 59.2) engaged in screen time, 24.9 min/day (SD = 29.5) using mobile screen devices, and 48.1 min/day (SD = 30.5) co-using with an adult. The majority of children’s screen time during the day was spent watching shows/movies/videos, and the majority of content was for entertainment purposes rather than educational purposes. The sensitivity analysis for total duration and patterns of children’s screen time for days parents recorded as being typical is presented in Table S1. On average, parents completed 11.5 typical days (SD = 2.2) of the two-week screen time diary. For almost all screen time variables, the mean was slightly lower for typical days in comparison to the full dataset that included atypical days.

**Table 3:** Patterns of Children’s Screen Time

	**Mean (SD) (*n* = 44)**
Total screen time (min/day)	103.5 (± 59.2)
Type	
Show/movie/video (min/day)	88.7 (± 56.8)
Electronic game (min/day)	7.3 (± 18.9)
Content	
Educational screen time (min/day)	14.2 (± 15.6)
Device	
Mobile device screen time (min/day)	24.9 (± 29.5)
Context	
Co-use (min/day)	48.1 (± 30.5)

The correlation coefficients between total duration and screen time patterns and cognitive development are presented in Table 4. Total screen time (*r*_*s*_ = -0.40; *p* = 0.011) and show/movie/video viewing (*r*_*s*_ = -0.42; *p* = 0.007) were significantly negatively correlated with working memory. The effect size for these correlation coefficients is considered medium [12]. Additionally, a medium effect size was observed for the negative association between co-use of traditional and mobile screen devices with self-control (*r*_*s*_ =—0.30; *p* = 0.057). The remaining correlations were all below *r*_*s*_ = 0.3 or a medium effect size. After adjusting for child age and parental education, medium effect sizes were still observed for total screen time (*r*_*s*_ = -0.32, *p* = 0.056), show/movie/video viewing *(r*_*s*_ = -0.32, *p* = 0.056) and working memory, though *p*-values were higher. Additionally, educational screen use was significantly positively correlated with vocabulary (*r*_*s*_ = 0.38,* p* = 0.018), while co-use was significantly negatively correlated with self-control (*r*_*s*_ = -0.32, *p* = 0.049), and these associations were represented by medium effect sizes. A medium effect size was also observed for the correlation between educational screen use and inhibitory control (*r*_*s*_ = 0.33, *p* = 0.074).

**Table 4:** Spearman Rho coefficients between diary-measures of screen time and cognitive development

	Total screen time (min/day)	Show/movie/ video(min/day)	Electronic game (min/day)	Educational screen use (min/day)	Mobile screen device use (min/day)	Co-use (min/day)
Bivariate Correlations						
Working memory (*n* = 40)	***r***_***s***_** = ** -0.40* ***p* = 0.011**	***r***_***s***_ = -0.42* ***p* = 0.007**	*r*_*s*_ = -0.28† *p* = 0.078	*r*_*s*_ = -0.09 *p* = 0.573	*r*_*s*_ = -0.05 *p* = 0.739	*r*_*s*_ = -0.19 *p* = 0.249
Inhibitory control (*n* = 34)	*r*_*s*_ = -0.16 *p* = 0.370	*r*_*s*_ = -0.28 *p* = 0.109	*r*_*s*_ = -0.06 *p* = 0.743	*r*_*s*_ = 0.15 *p* = 0.382	*r*_*s*_ = 0.23 *p* = 0.189	*r*_*s*_ = -0.18 *p* = 0.299
Vocabulary (*n* = 42)	*r*_*s*_ = -0.21 *p* = 0.189	*r*_*s*_ = -0.29† *p* = 0.066	*r*_*s*_ = 0.22 *p* = 0.166	*r*_*s*_ = 0.24 *p* = 0.121	*r*_*s*_ = 0.20 *p* = 0.214	*r*_*s*_ = 0.02 *p* = 0.901
Self-control (*n* = 41)	*r*_*s*_ = 0.03 *p* = 0.867	*r*_*s*_ = -0.03 *p* = 0.867	*r*_*s*_ = 0.06 *p* = 0.723	*r*_*s*_ = 0.07 *p* = 0.678	*r*_*s*_ = 0.15 *p* = 0.341	*r*_*s*_ = -0.30 *p* = 0.057†
Partial Correlations^a^						
Working memory (*n* = 40)	*r*_*s*_ = -0.32 *p* = 0.056†	*r*_*s*_ = -0.32 *p* = 0.056†	*r*_*s*_ = -0.26 *p* = 0.125	*r*_*s*_ = 0.10 *p* = 0.574	*r*_*s*_ = -0.03 *p* = 0.876	*r*_*s*_ = -0.14 *p* = 0.410
Inhibitory control (*n* = 34)	*r*_*s*_ = -0.13 *p* = 0.496	*r*_*s*_ = -0.20 *p* = 0.276	*r*_*s*_ = -0.13 *p* = 0.499	*r*_*s*_ = 0.33 *p* = 0.074†	*r*_*s*_ = 0.26 *p* = 0.155	*r*_*s*_ = -0.19 *p* = 0.304
Vocabulary (*n* = 42)	*r*_*s*_ = -0.20 *p* = 0.213	*r*_*s*_ = -0.27 *p* = 0.102	*r*_*s*_ = 0.23 *p* = 0.156	***r***_***s***_ = 0.38* ***p* = 0.018**	*r*_*s*_ = 0.21 *p* = 0.204	*r*_*s*_ = 0.08 *p* = 0.644
Self-control (*n* = 41)	*r*_*s*_ = 0.04 *p* = 0.815	*r*_*s*_ = 0.01 *p* = 0.948	*r*_*s*_ = 0.05 *p* = 0.746	*r*_*s*_ = 0.16 *p* = 0.349	*r*_*s*_ = 0.19 *p* = 0.265	***r***_***s***_ = -0.32* ***p* = 0.049**

^a^ Partial correlations are adjusted for child age and parental education

^*^p < 0.05; †*p* < 0.10

The sensitivity analysis for the correlation coefficients between total duration of screen time, patterns of screen time, and cognitive development on typical days only are presented in Table S2. Similar to the main analysis, medium effect sizes were observed for total screen time and show/movie/video viewing and working memory. However, unlike the main analysis, a medium effect size was not observed for co-use and self-control (*r*_*s*_ = -0.28,* p* = 0.092). After adjusting for child age and parental education, medium effect sizes for total screen time and working memory (*r*_*s*_ = -0.30, *p* = 0.073) and educational screen time and inhibitory control (*r*_*s*_ = -0.30,* p* = 0.089) were observed but they were no longer significant. Additionally, educational screen use was significantly positively correlated with vocabulary (*r*_*s*_ = 0.32, *p* = 0.049). Unlike the main analysis, a medium effect size was not observed for the correlation between show/movie video viewing and working memory.

The summary of the parent**–**child interaction scores between the three tasks are presented in Table 5. The results showed that there was a statistically significant difference in parent**–**child interaction quality scores for the three tasks (*p* < 0.001). The Bonferroni contrast revealed that the parent**–**child interaction quality significantly differed between the video and storybook reading tasks (*p* < 0.001, *d* = 0.70), the video and electronic game tasks (*p* = 0.003, *d* = *2.*56), and the storybook reading and electronic game tasks (*p* < 0.001, *d* = 1.48). Medium and large effect sizes were observed for the difference in parent–child interaction quality between the three tasks. The video viewing task had the lowest parent**–**child interaction quality average score (*M* = 31.70, *SD* = 4.14) and the electronic game had the highest average score (*M* = 43.39, SD = 4.96).

**Table 5:** Summary of parent–child interaction quality scores

Task	N	*M*^a^	*SD*
Video	42	31.70	4.14
Electronic game	42	43.39	4.96
Storybook reading	42	35.30	5.92

^a^ The scores for all three tasks were significantly different

## Discussion

This pilot study assessed the feasibility of a virtual study protocol examining preschool children’s screen time, cognitive development, and interactions with parents between three tasks. The results of this study indicate that this virtual study protocol is feasible for conducting a future longitudinal study on the association between screen time and cognitive development, though some minor modifications may be needed. Specifically, in terms of eligibility, children will also need to speak fluently in English to enable the scoring of parent–child interaction tasks. Additionally, given the number of children that did not complete the HTKS test, procedures for keeping children engaged as well as procedures for scoring the HTKS test when a child does not want to finish, may need to be considered for a future study.

This pilot study also addressed current gaps in the literature by examining preliminary hypotheses of association for patterns of preschool children’s screen time use and its association with various domains of cognitive development (i.e., memory, executive functioning, and language) among preschool children aged 3 years, as well as whether the quality of parent–child interactions differed between three tasks. Based on parental daily diary reports, show/movie/video viewing, referred to as video viewing hereafter, was the most common type of screen time and parent–child interactions during video viewing were of the lowest quality. In contrast, electronic game use was the least common type of screen time, but it had significantly higher parent–child interaction quality compared to video and storybook reading tasks. Some correlations with medium effect sizes were observed, though the direction of correlations differed based on the screen time variable and the cognitive developmental outcome. Total duration of screen time, video viewing, and co-use were negatively associated with some cognitive developmental measures, whereas educational screen time was positively associated with vocabulary.

A novel aspect of the present study was examining the patterns of preschool children’s screen time measured via an online daily diary design. The results of the pilot study indicate that an online 2-week daily diary design is feasible to collect data on the patterns of preschool children’s screen time. Previous studies have primarily relied on the use of parent questionnaires to measure children’s screen time habits, typically by focusing on the total duration and/or frequency of children’s screen time [8]. Only one recent study to our knowledge used a daily screen time diary to examine children’s media quantity, content, and context in Saudi children aged 1 to 3 years [2]. Our finding regarding the high prevalence of watching videos on traditional or mobile screen devices for entertainment purposes is consistent with the findings of this study. Specifically, children spent the majority of screen time watching child-directed non-educational content on all screens [2]. These findings suggest that while mobile media devices can be interactive and used for a variety of activities, it appears young children may primarily use these screen devices passively. This finding may be the result of parents using screens to entertain or occupy children while they complete other tasks as interactive screen time may require more parental engagement and support [7]. Thus, future research building on this work should capture parent’s intent for each screen time session.

It is important to note that some content categorized as entertainment in the present study was marketed as educational. This is in line with a recent study that found 58% of apps labeled as educational for young children were low-quality on a coding scheme assessing active learning, engagement in learning, meaningful learning, and social interaction [32]. One study examining parent perceptions of the risks and benefits of screen time found that 82% of parents believed that screen time provided opportunities for learning and education [23]. Therefore, the potential disconnect between what parents perceive as educational, potentially due to marketing, and what programs and apps are actually of high-quality for educational or learning outcomes should be explored further. Potentially the diary used for this study could also ask parents to categorize the content along with recording the content for researchers to later categorize.

The present study found that educational screen time, as defined by researchers, was significantly positively correlated with vocabulary, even after adjustment of covariates and the removal of non-typical diary days. This finding is consistent with a recent meta-analysis that found educational television programs were significantly associated with stronger language skills in children 12 years and younger [30, 31]. Unique to the present study was the inclusion of electronic games in addition to videos in the educational screen time variable. Our findings for total screen time, video viewing, and working memory align with the findings from a large systematic review that found primarily null or detrimental effects of screen time on various domains of cognitive development [9]. Since findings of the current study suggest associations with screen time may differ across these domains, future studies should examine the associations between screen time and cognitive development across language, memory, and executive function domains in larger more generalizable samples. Additionally, future studies should consider the patterns of screen time, not just the total duration, to better understand the impacts of screen time on these domains of cognitive development.

One potential mechanism to explain why excessive screen time may be negatively associated with children’s cognitive development is through the displacement of interactions with caregivers, such as displacing non-screen-based language and play based interactions with screen time [38]. High-quality parent–child interactions are important for optimal growth and development in early childhood. Co-use of screen devices may mitigate some of the negative impacts of screen time by providing opportunities for parent–child interactions, scaffolding, and feedback [33, 44]. However, in the present study co-use was negatively associated with self-control. This may be explained by the fact that video viewing was the most prevalent type of screen time, and video viewing had the lowest quality of parent–child interactions. In contrast, co-use of electronic games had the highest quality of parent–child interactions, but this type of screen time was not prevalent among young children. Taken together, these findings suggest future research should take into account that quality of parent–child interactions during co-use may be more important than the quantity of co-use.

One major strength of this study was the use of the daily screen time diary to measure the patterns of children’s screen time. Screen time daily diaries allow for more a comprehensive measure of children’s screen time [8]. One limitation is the small sample size, given this was a pilot study, therefore, the results may not be generalizable to all children aged 3 years in Canada. While the race/ethnicity composition of our sample is consistent with the Alberta, Canada population [42], future studies should aim to recruit a sample that reflects the diversity in social-economic status. Additionally, residual confounding may have occurred due to unmeasured variables (e.g., parent availability, parental stress). For the parent–child interaction tasks, families used a variety of devices for completing the tasks (i.e., desktop computer, laptop, tablet). Future research should consider standardizing the device for the electronic game task to a mobile device (e.g., smartphone) as these devices may be more familiar to children and better represent the device they would use for electronic games in everyday life [4]. Additionally, previous research has found that parent–child interactions can differ between printed books and e-books [27]. Therefore, the differences observed for the quality of parent**–**child interactions may not be generalizable to printed books and should be confirmed with printed books in future research. Finally, it is important to note that the cognitive development tests used in this study were validated for in-person assessments not virtual assessments, however the data for this study were collected virtually due to the COVID-19 pandemic and previous research has shown remote assessments of executive functioning, which have been converted from in-person assessments, to be reliable and valid among preschoolers [1].

## Conclusion

Overall, the findings of this study suggest that the virtual study protocol was feasible. Additionally, the findings suggest that excessive screen time may be detrimental for some domains of cognitive development, but the type, content, and context is important to consider in future studies with daily diaries. In particular, high-quality educational screen use may have some cognitive development benefits, especially for language development, but it may be difficult for parents to identify high-quality versus low-quality programming and electronic games. Additionally, while interactive activities, such as playing electronic games and reading storybooks had higher quality parent–child interactions in comparison to television viewing, children primarily use traditional and mobile screen devices for show/movie/video viewing. A future longitudinal study, using this virtual protocol, will be conducted with a larger more generalizable sample to test these findings for replication and examine age-related change.

## Supplementary Information

**Additional file 1: Table S1. **Patterns of children’s screen time with typical days only.** Table S2. **Spearman Rho coefficients between diary-measures of screen time for typical days only and cognitive development.

## Data Availability

The data that support the findings of this study are available on reasonable request from the corresponding author. The data are not publicly available due to privacy or ethical restrictions.
