# Supplementary material for: Patterns of preschool children’s screen time, parent–child interactions, and cognitive development in early childhood: a pilot study
Source: Pilot Feasibility Stud. 2023 Mar 14;9:39. doi: 10.1186/s40814-023-01266-6 (PMC10012297; doi:10.1186/s40814-023-01266-6)
Supplement: Supplementary file 1 — Additional file 1: Table S1. Patterns of children’s screen time with typical days only. Table S2. Spearman Rho coefficients between diary-measures of screen time for typical days only and cognitive development. [file 40814_2023_1266_MOESM1_ESM.docx]

**Table S1**

*Patterns of children’s screen time with typical days only*

|  | **Mean (SD) (n = 44)** |
| --- | --- |
|  |  |
| Total screen time (min/day) | 99 (± 60.7) |
| Type |  |
| Show/movie/video (min/day) | 85.2 (± 59.0) |
| Electronic game (min/day) | 7.3 (±18.9) |
| Content |  |
| Educational screen time (min/day) | 13.9 (±16.1) |
| Device |  |
| Mobile device screen time (min/day) | 24.7 (±31.5) |
| Context |  |
| Co-use (min/day) | 45.6 (± 31.6) |

**Table S2**

*Spearman Rho coefficients between diary-measures of screen time for typical days only and cognitive development*

|  | Total screen time (min/day) | Show/movie/Video (min/day) | Electronic game (min/day) | Educational screen use (min/day) | Mobile screen device use (min/day) | Co-use (min/day) |
| --- | --- | --- | --- | --- | --- | --- |
| Bivariate  Correlations |  |  |  |  |  |  |
| Working memory (n=40) | ***r_s_* =-0.39***  ***p* = 0.013** | ***r_s_* = -0.40***  ***p* = 0.012** | *r_s_* = -0.26  *p* = 0.108 | *r_s_* = -0.04  *p* = 0.810 | *r_s_* = 0.00  *p* = 0.989 | *r_s_* = -0.22  *p* = 0.178 |
|  |  |  |  |  |  |  |
| Inhibitory control (n=34) | *r_s_* = -0.15  *p* = 0.389 | *r_s_* = -0.24  *p* = 0.168 | *r_s_* = -0.03  *p* = 0.859 | *r_s_* = 0.22  *p* = 0.213 | *r_s_* = 0.28  *p* = 0.110 | *r_s_* = -0.05  *p* = 0.775 |
|  |  |  |  |  |  |  |
| Vocabulary (n=42) | *r_s_* = -0.16  *p* = 0.317 | *r_s_* = -0.25  *p* = 0.118 | *r_s_* = 0.19  *p* = 0.234 | *r_s_* = 0.23  *p* = 0.142 | *r_s_* = 0.25  *p* = 0.105 | *r_s_* = -0.01  *p* = 0.947 |
|  |  |  |  |  |  |  |
| Self-control (n=41) | *r_s_* = 0.04  *p* = 0.785 | *r_s_* = -0.02  *p* = 0.913 | *r_s_* = 0.02  *p* = 0.908 | *r_s_* = 0.12  *p* = 0.439 | *r_s_* = 0.20  *p* = 0.214 | *r_s_* = -0.24  *p* = 0.134 |
| Partial  Correlations^a^ |  |  |  |  |  |  |
| Working memory (n=40) | *r_s_*=-0.30  *p* = 0.073† | *r_s_*=-0.28  *p* = 0.089† | *r_s_*=-0.28  *p* = 0.098 | *r_s_*=0.079  *p* = 0.644 | *r_s_*= -0.035  *p* = 0.983 | *r_s_*=-0.19  *p* = 0.254 |
| Inhibitory control (n=34) | *r_s_*=-0.10  *p* = 0.608 | *r_s_*=-0.14  *p* = 0.462 | *r_s_*= -0.09  *p* = 0.627 | *r_s_*=0.30  *p* = 0.097 | *r_s_*=0.29  *p* = 0.114 | *r_s_*=-0.08  *p* = 0.680 |
| Vocabulary (n=42) | *r_s_*=-0.12  *p* = 0.457 | *r_s_*=-0.21  *p* = 0.201 | *r_s_* = 0.17  *p* = 0.297 | ***r_s_*=0.32***  ***p* = 0.049** | *r_s_*=0.24  *p* = 0.137 | *r_s_=*0.02  *p* = 0.889 |
| Self-control (n=41) | *r_s_*=0.07  *p* = 0.658 | *r_s_*=0.04  *p* = 0.831 | *r_s_*=-0.01  *p* = 0.976 | *r_s_*=0.17  *p* = 0.310 | *r_s_*=0.21  *p* = 0.206 | *r_s_*=-0.28  *p* = 0.092† |

***p<0.05**; †p<0.10

Note: ^a^ Partial correlations are adjusted for child age and parental education
